# Automatic depression severity assessment with deep learning using parameter-efficient tuning

**DOI:** 10.3389/fpsyt.2023.1160291

**Published:** 2023-06-15

**Authors:** Clinton Lau, Xiaodan Zhu, Wai-Yip Chan

**Affiliations:** Department of Electrical and Computer Engineering & Ingenuity Labs, Queen's University, Kingston, ON, Canada

**Keywords:** depression assessment, prefix-tuning, transfer learning, deep learning, clinical decision support, natural language processing (NLP)

## Abstract

**Introduction:**

To assist mental health care providers with the assessment of depression, research to develop a standardized, accessible, and non-invasive technique has garnered considerable attention. Our study focuses on the application of deep learning models for automatic assessment of depression severity based on clinical interview transcriptions. Despite the recent success of deep learning, the lack of large-scale high-quality datasets is a major performance bottleneck for many mental health applications.

**Methods:**

A novel approach is proposed to address the data scarcity problem for depression assessment. It leverages both pretrained large language models and parameter-efficient tuning techniques. The approach is built upon adapting a small set of tunable parameters, known as prefix vectors, to guide a pretrained model towards predicting the Patient Health Questionnaire (PHQ)-8 score of a person. Experiments were conducted on the Distress Analysis Interview Corpus - Wizard of Oz (DAIC-WOZ) benchmark dataset with 189 subjects, partitioned into training, development, and test sets. Model learning was done on the training set. Prediction performance mean and standard deviation of each model, with five randomly-initialized runs, were reported on the development set. Finally, optimized models were evaluated on the test set.

**Results:**

The proposed model with prefix vectors outperformed all previously published methods, including models which utilized multiple types of data modalities, and achieved the best reported performance on the test set of DAIC-WOZ with a root mean square error of 4.67 and a mean absolute error of 3.80 on the PHQ-8 scale. Compared to conventionally fine-tuned baseline models, prefix-enhanced models were less prone to overfitting by using far fewer training parameters (<6% relatively).

**Discussion:**

While transfer learning through pretrained large language models can provide a good starting point for downstream learning, prefix vectors can further adapt the pretrained models effectively to the depression assessment task by only adjusting a small number of parameters. The improvement is in part due to the fine-grain flexibility of prefix vector size in adjusting the model's learning capacity. Our results provide evidence that prefix-tuning can be a useful approach in developing tools for automatic depression assessment.

## 1. Introduction

Major depressive disorder is a common psychiatric disorder affecting over 264 million people worldwide (1). It has profound impact on a patient's emotional and physical health, and also the individual's family's quality of life. By 2030, depression is expected to be the second leading cause of disease burden in the world (2). Early recognition of the illness is imperative to promote remission and reduce the emotional and financial burden of the disease (3). The common challenges humans need to face, such as pandemics, can often worsen the situation. It has been estimated that the COVID-19 pandemic and its associated public health and social measures have led to a 27.6% increase in cases of major depressive disorder (4). In 2021, a survey reported 72% of psychologists had experienced a rapid rise in demand for treatment of depressive disorder, alongside with increased workloads, longer waitlists, and low capacity for new patients (5). To help ease the burden on the rest of the healthcare system, early in the COVID-19 pandemic the United States Food and Drug Administration relaxed premarket requirements for mobile health therapeutic apps that treat psychiatric conditions (6).

Recently, machine learning (ML) methods have become a point of interest in supporting mental health providers for decision-making (e.g., diagnosis, prognosis, treatment, etc.) based on a plethora of clinical data, including vocal and visual expression data (7). ML-powered technologies could help offload the burden for psychologists by providing standardized assessment processes. ML competitions have facilitated the acceleration of research in developing new tools to support precise depression diagnosis, with one particular series focusing on the prediction of depression severity through clinical interviews (8–10).

Deep learning (DL) is one of the sub-fields of ML that has seen significant advancement over the past decade across almost all research fields and application-driven tasks, including healthcare (11). The impressive performance of deep neural networks can be attributed to the increase in available training data and the expressiveness of these networks, which is often marked by large numbers of model parameters. However, one key challenge in many healthcare applications, including depression and Alzheimer's disease recognition, has been the limited amount of training data (7), typically at the level of a few hundred samples or sometimes even less (9, 12–14). Cost and privacy concerns further pose challenges for large-scale collection of medical data.

A general approach to solving the challenge of data scarcity has been transfer learning through pretrained DL models. This approach typically involves a neural network first learning to solve a source task, from which knowledge acquired is stored as model parameters to be transferred to solve a separate target task. Deep transfer learning has the advantage of speeding up training by using far fewer target-task training data to achieve good performance. By the same token, it allows a model to achieve good performance when training data of the target task is limited. Intuitively, a pretrained DL model has learned sufficiently general representation for a data domain (e.g., language, speech, and vision) through supervised or self-supervised training that utilizes a large amount of generic data. Then, the pretrained model can be re-purposed for a target task. The recent advancement in pretrained language models (e.g., BERT (15), GPT (16) and their more recent variants) can be seen as a specific but foundational form of transfer learning, which often improves the performance on target tasks by leveraging large-scale datasets during pretraining. The two main strategies for doing transfer learning with a pretrained model are (1) using it directly without any modifications to the parameters (e.g., in-context learning) and (2) further adapting it on the target data.

As the most typical approach, *fine-tuning*, which involves tuning pretrained parameters on a target dataset, has been the de facto practice of adapting pretrained models to the target task. It aims to improve the performance of pretrained models by learning information specific to the target domain. To reduce the effect of overfitting when training data is limited, the number of fine-tuning parameters is reduced by restricting tuning to a subset of layers, often the layers closest to the output with higher-order representations (17). Consequently, deep transfer learning is aptly suitable for training ML models on depression data. The work presented in Zhao et al. (18) applied transfer learning by using hierarchical attention models. It pretrained parameters of their attention mechanism on a speech recognition dataset before transferring them to their final model for fine-tuning on depression speech data. Another work pretrained an emotion recognition model on two emotion datasets before further adapting it to depression data (19). Instead of pretraining from scratch, prior work have also leveraged pretrained large language models and fine-tuned them on their respective target depression datasets (20, 21).

Despite prior success in adapting pretrained language models to depression data by fine-tuning, it has a major drawback. The number of parameters to be fine-tuned is determined by the pretrained model architecture. For instance, BERT is typically fine-tuned by at least a layer with over seven million parameters (22), which is excessive for less than a few hundred training samples. This problem is further exacerbated by the rapid growth in the size of pretrained language models. Fine-tuning large models with a small amount of data is a recipe for overfitting (23), as with the case of depression data. In this paper, we explore an alternative means of model adaptation by leveraging the most recent advancements on parameter-efficient tuning. We propose applying prefix-tuning (24) for the adaptation of pretrained large language models on depression data. As an ultra-lightweight alternative to fine-tuning, the prefix-tuning adaptation method works by prepending a small number of trainable continuous vectors to a deep network, i.e., every layer of a pretrained language model. During training, model parameter updates are only performed on the prepended vectors while the pretrained model parameters remain frozen. This has an advantage of decoupling the number of tuning parameters from the model architecture itself and therefore allows flexible adjustment to the model's learning capacity. Motivated by recent work suggesting that the lightweight parameter footprint of prefix-tuning is less likely to overfit than fine-tuning (24, 25), we adapted pretrained language encoders to the task of depression analysis using prefix vectors. To the best of our knowledge, this work is the first instance of leveraging prefix-tuning for the task of depression diagnosis prediction.

Our experimental results on the publicly available *Distress Analysis Interview Corpus—Wizard of Oz* (DAIC-WOZ) benchmark dataset showed that prefix-tuned models outperformed conventional fine-tuning approaches, as well as models utilizing multiple modalities reported in the current literature. Furthermore, when embeddings extracted from a depression-adapted encoder and a general-purpose encoder were combined, we achieved the best performance for depression severity prediction reported so far on DAIC-WOZ. Additional experiments revealed the ability of prefix-based models to perform better than fine-tuning based models. Moreover, we studied the effects of training set size and the results suggested that the prefix-tuning method was more capable for low-data settings than fine-tuning methods. Our proposed method achieved a new state-of-the-art performance on the DAIC-WOZ. Results from this study show promise in applying machine learning to automatically assist care providers with depression assessment.

## 2. Materials and methods

### 2.1. DAIC-WOZ dataset

**Figure 1:**
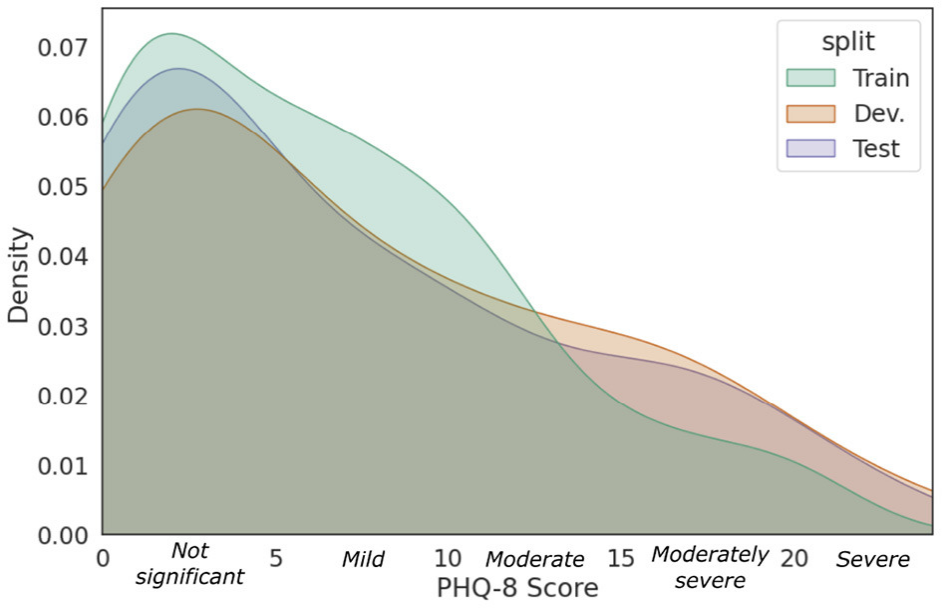
Data distribution of DAIC-WOZ with respect to the PHQ-8 scores of the training, development, and test sets.

DAIC-WOZ (26) is a commonly-used benchmark dataset for experimenting and validating ML models for automatizing depression assessment. The dataset consists of clinical interviews with 189 subjects, partitioned into a training set (57%, 107 subjects), a development set (19%, 35 subjects), and a test set (25%, 47 subjects). The interviews were conducted by an animated virtual interviewer named Ellie, which was remotely controlled by two clinicians in a separate room to select appropriate questions, responses, and gestures in real-time. During an interview, questions asked were selected from a predefined set of questions, while the selections were determined in real-time based on the context of the conversation. Thus, each interview had a unique set of questions asked. Nevertheless, the interviews were semi-structured. They began with rapport-building questions, such as inquiring the subject's background, hobbies, and interests. Thereafter, questions related to symptoms of depression such as their recent sleep pattern and mood were posed. When a significant personal event was brought up, the subject was asked to introspect and describe their emotional state at that instance. They were also asked to state any previous history with depression and PTSD and Ellie would invite them to detail their recovery progress when stated. All interviews concluded with a set of cool-down questions to allow the subjects to come to ease before they leave their sessions. Each subject was assigned to complete an eight-item Patient Health Questionnaire (PHQ-8) prior to his/her interview. Each item on the questionnaire is scored from 0 points if the condition is absent, to three points, if severe (27). The eight scores are summed to form the PHQ-8 score which ranges from 0 to 24 and serves as an estimate of the individual's depression severity. The resulting PHQ-8 score was used as the ground-truth label for depression severity. The total score can be further categorized into five severity ranges. Scores of 5, 10, 15, and 20 represent cutpoints for mild, moderate, moderately severe, and severe depression, respectively. The objective of our study is to predict the total PHQ-8 score as a proxy for the participant's depression severity. Moreover, transcriptions of the spoken dialogues between the interviewer and subject were provided, which include verbal and non-verbal annotations such as acronyms, sighs, coughs, and laughter. Raw audio recordings and visual-based features were also provided but were not utilized in this study. Figure 1 shows the kernel density estimates of the PHQ-8 distributions between the three data partitions. We observe that the PHQ distributions of the partitions are right-skewed, which resembles the real-world distribution of PHQ scores (28). The skewness is maintained as it is desirable for the data distributions of the validation and test sets to be reflective of the real-world data.

### 2.2. Problem formulation

We formalize the problem of using a ML model to predict the depressive state of an interview subject via spoken text of conversational dialogue. Formulated as a supervised learning task, the model is trained to predict the PHQ-8 score y∈Y from an input interview transcription x∈X, from training data. As the PHQ-8 score takes on an integer value from 0 to 24, we formulate the task as a regression problem. We regard a conversational interview as a semi-structured dialogue between a clinician and a subject, from whom the clinician elicits information insightful to the subject's state of mind through a series of questions. A pattern can be identified in which the clinician asks a question *q*, followed by a response *r* from the subject. We group each question and response together to model *x* as a sequence of *N* question-response (QR) pairs, *S* = [*s*_1_, …, *s*_*N*_], where *s*_*n*_ is a concatenation of *q*_*n*_ and *r*_*n*_ for *n*∈{1, …, *N*}.

**Figure 2:**
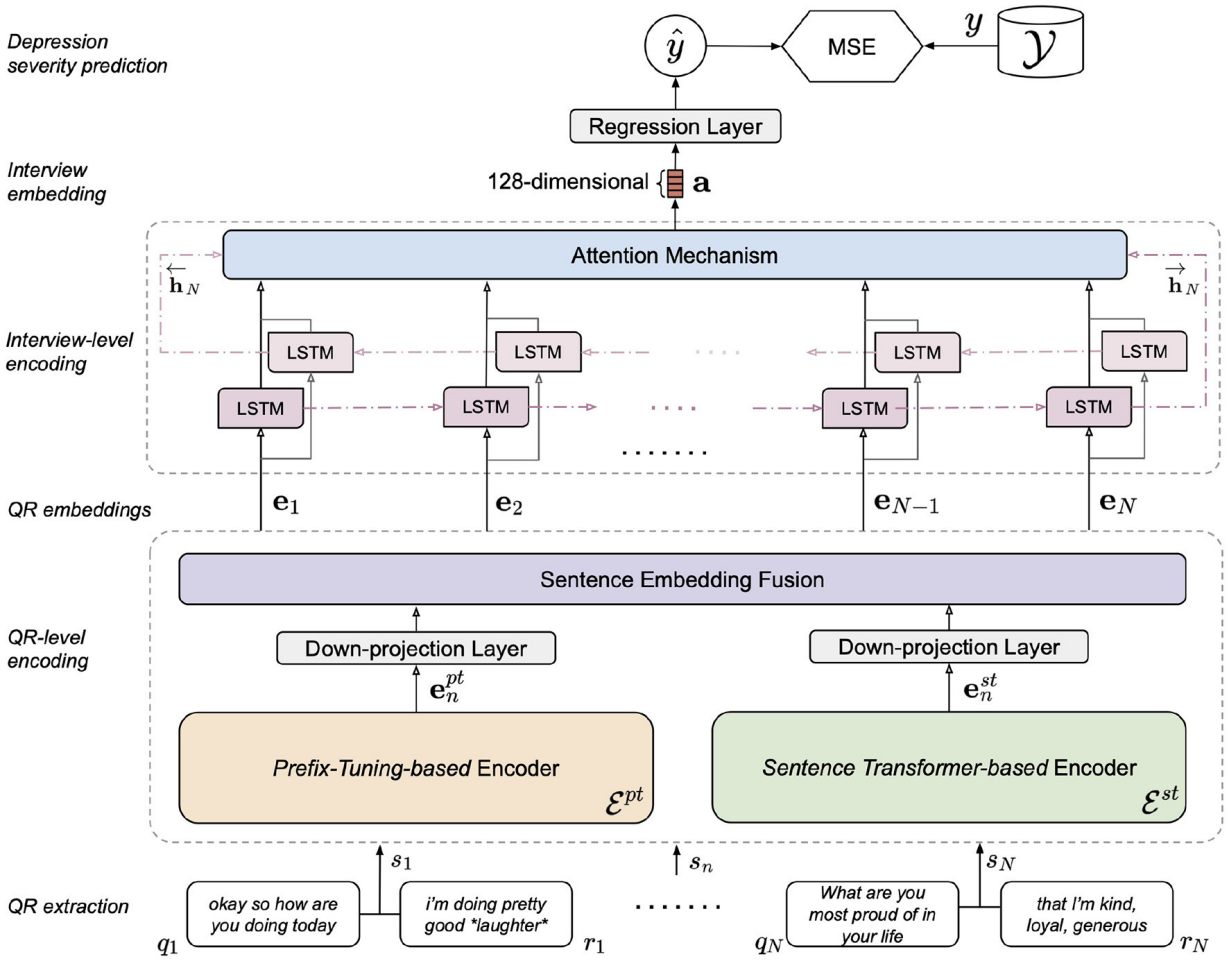
Overview of the proposed dual encoder model architecture.

As illustrated in Figure 2, our model encodes *S* in two stages. In the first stage, a language encoder embeds each variable-length QR pair *s*_*n*_ as a fixed-length numeric vector (embedding) **e**_*n*_, such that *S* is mapped to a sequence of QR-level embeddings *E* = [**e**_1_, …, **e**_*N*_]. Ideally, **e**_*n*_ encapsulates the semantic and contextual meaning of *s*_*n*_, of which the quality depends on the language encoder used. We describe our proposed approach for QR-level encoding in Section 2.3. In the second stage, a sequence encoder encodes *E* as a fixed-length interview-level embedding **a**. Conceptually, **a** summarizes the entire interview. Section 2.4 details the implementation of our interview-level sequence encoder. Finally, **a** is passed through a linear layer to output a PHQ-8 score prediction ŷ.

### 2.3. Question-response-level modeling: prefix-tuning + sentence transformer

We explore two approaches for the QR-level encoding. In the first approach, as described in Section 2.3.1, we discuss adapting pretrained language encoders to our target depression dataset by prefix-tuning. This model adaptation method has been shown to achieve comparable performance to traditional fine-tuning method while using significantly less trainable model parameters. A model with larger learning capacity (more learnable parameters) is more prone to overfitting, wherein the trained model performs well on the training data but generalizes poorly on unseen data. The risk of overfitting is magnified when training data is scarce. We hypothesize that depression-adapted embeddings extracted via prefix-tuning are more suitable than embeddings from fine-tuning. In the second approach, as described in Section 2.3.2, we discuss the use of pretrained encoders without modifying the pretrained weights to suit our limited training data. Pretrained embeddings can be seen as general-purpose without any task-specific knowledge. We aim to show that by carefully selecting an appropriate pretrained encoder, it can extract effective QR embeddings. Furthermore, we hypothesize that depression-adapted and general-purpose embeddings are complementary with one another. In Section 2.3.3, we describe our experimentation with fusing both types of embedding.

#### 2.3.1. Depression-adapted encoding via prefix-tuning

Performance envelope advancements from pretrained large language models can be partly attributed to their continuing growth in size. While application-driven researchers are disposed to adopt the newest models to their specific needs, the number of learnable parameters often grows much faster than application-specific data available for model adaptation, e.g., just one BERT transformer (29) layer has ~7M parameters. Although prior work have found success on fine-tuning pretrained language models for specific tasks, we posit these models are overparameterized for specialized tasks and thus provide room for as yet unrealized potential performance gain.

Initially conceived as a more efficient and effective method to extract information from pretrained models, prefix-based methods have shown remarkable results on a wide range of downstream tasks, including conditional generation tasks and sequence labeling tasks (24, 25, 30). Specifically, prefix-tuning (24) works by introducing a small set of trainable continuous vectors to a pretrained transformer-based model and only updates the prefix vectors while keeping all the pretrained weights frozen. Originally developed for natural language generation tasks, we retrofit prefix-tuning for feature extraction on QR pairs.

**Figure 3:**
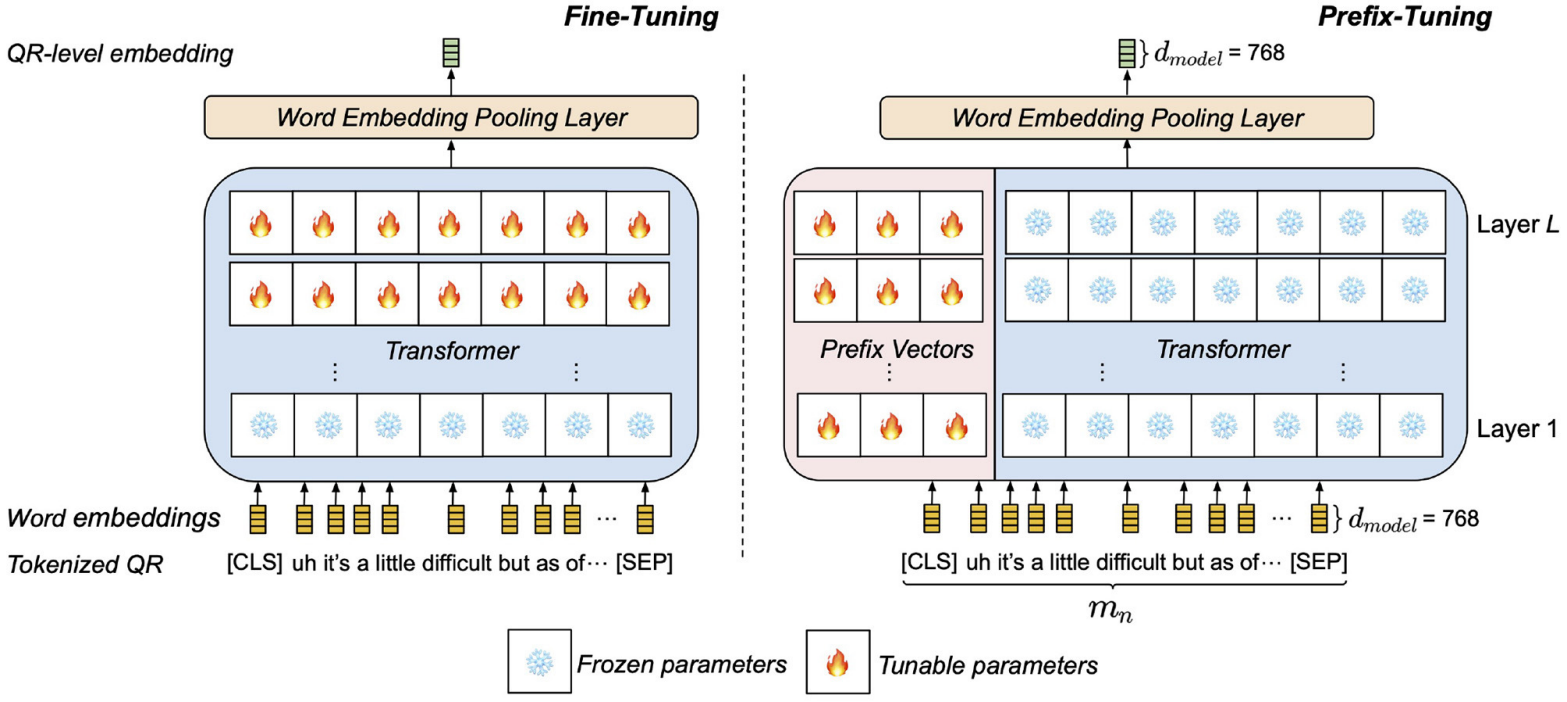
High-level comparison between the two depression adaptation methods of QR encoding on an example tokenized QR pair. **(Left)** QR encoding by fine-tuning pretrained layers. **(Right)** QR encoding by prepending prefix vectors to pretrained layers.

Our prefix-tuning-based QR encoder $E^{pt}$ assumes a pretrained language encoder based on the transformer architecture (e.g., BERT, RoBERTa (31)) with *L* layers and hidden size *d*_*model*_. Each transformer layer *l*∈{1, …, *L*} contains *I*_*head*_ parallel self-attention layers (heads), which allow word-level embeddings in an input sequence to draw dependencies between each other. To adapt the pretrained encoder to the depression data, task-specific prefix vectors are prepended to every key-value pair of self-attention as learnable parameters. As illustrated in Figure 3, while fine-tuning modifies pretrained parameters near the output, tunable prefix vectors are added to every layer, a mechanism found to engender more expressive adaptation. In contrast to fine-tuning, prefix-tuning freezes the core pretrained model parameters and only modifies the auxiliary set of task-specific parameters. This prevents the model from altering the general comprehension of language acquired from pretraining (25).

The details of the transformer's self-attention can be found in Vaswani et al. (29). For completeness of this paper and for facilitating the discussion of our prefix-tuning-based components, we summarize the central idea of the self-attention mechanism below. Given a self-attention head that takes as input a sequence of *m*_*n*_ word-level embeddings ${\text{S}}_{n}\in {\mathbb{R}}^{m_{n}\times d_{model}}$, the head projects **S**_*n*_ to queries, keys, and values $\text{Q},\text{K},\text{V}\in {\mathbb{R}}^{m_{n}\times d_{head}}$ through pretrained matrices ${\text{W}}^{Q},{\text{W}}^{K},{\text{W}}^{V}\in {\mathbb{R}}^{d_{model}\times d_{head}}$, respectively, where $d_{head}=\frac{d_{model}}{I_{head}}\in \mathbb{N}$:^1^


(1) $$\begin{array}{l} \text{head}=\text{Attention}(S_{n}W^{Q},S_{n}W^{K},S_{n}W^{V})\\ \;=\text{Attention}(Q,K,V)\\ \;=\text{Softmax}(\frac{QK^{⊤}}{\sqrt{d_{head}}})V \end{array}$$

In practice, multiple self-attention heads parameterized by ${\text{W}}_{l,i}^{\left\{Q,K,V\right\}}$ work in parallel for *i*∈{1, …, *I*_*head*_}. Prefix-tuning introduces two sets of prefix vectors ${\text{P}}^{K},{\text{P}}^{V}\in {\mathbb{R}}^{|P|\times d_{head}}$ to each head with |*P*| being the prefix vector's length. By prepending them to the keys and values, Equation (1) becomes:


(2) $$\begin{array}{l} \text{head}=\text{Attention}(Q,[P^{K};K],[P^{V};V])\\ \;=\text{Softmax}(\frac{Q{\left[P^{K};K\right]}^{⊤}}{\sqrt{d_{head}}})[P^{V};V] \end{array}$$

where the semicolon ; denotes concatenation. The set of all prefix vectors is randomly initialized and gathered as a tensor $\text{P}\in {\mathbb{R}}^{L\times I_{head}\times |P|\times d_{head}\times 2}$.

**Figure 4:**
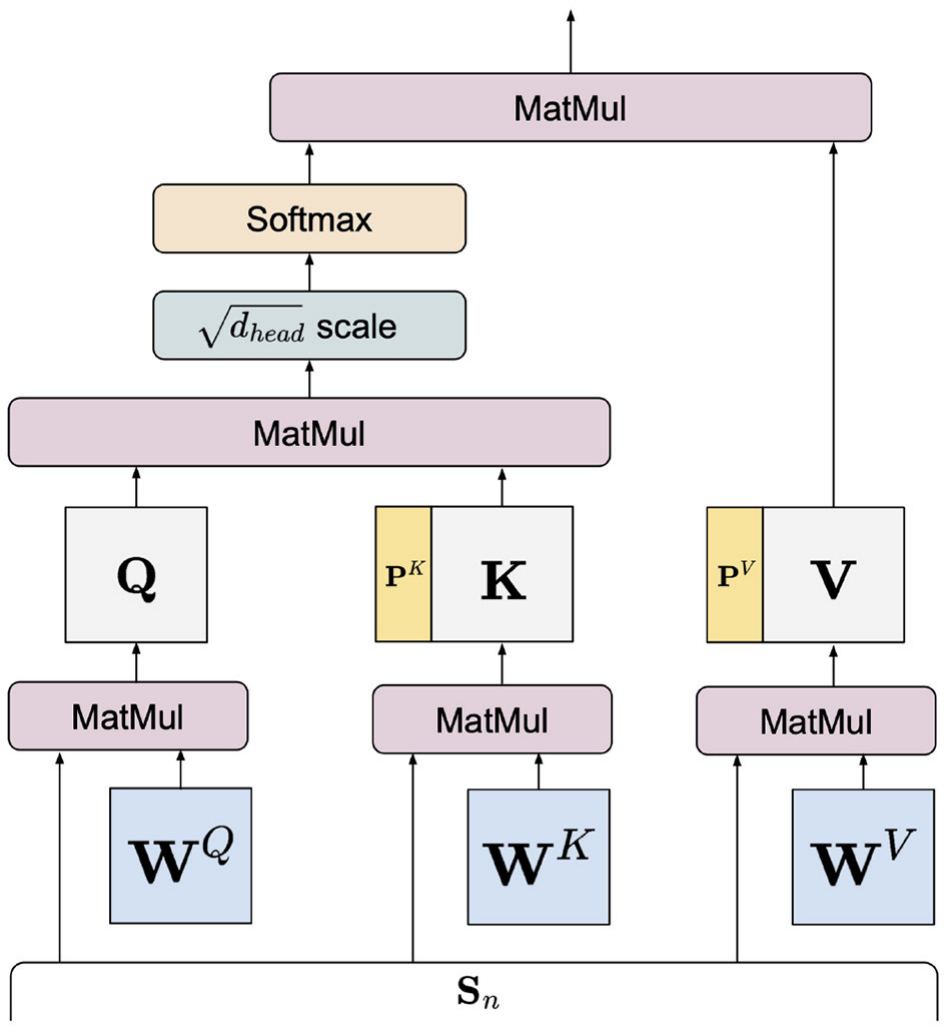
A prefix-enhanced self-attention head.

We manifest Equation (2) as Figure 4 to graphically illustrate the insertion mechanism of prefix vectors in a self-attention layer. These prefix-enhanced self-attention layers are only present in the transformer layers of $E^{pt}$ in Figure 2. In self-attention, keys and queries are essentially used to compute self-attention weights for the values. These prefix-enhanced keys and values are propagated up through the transformer and error is back-propagated through **P** while all other encoder parameters are kept frozen. Effectively, once tuning is completed, the fixed-value **P** plays a similar role to the pretrained parameters ${\text{W}}_{l,i}^{\left\{K,V\right\}}$, such that inserting **P** into the pretrained encoder effectively modulates the distribution of the original pretrained parameters in mapping the input embeddings. Compared to fine-tuning, adaptation via prefix vectors is localized at the keys and values while its effect permeates throughout the whole network. Furthermore, the prefix length |*P*| being a design hyperparameter allows a more flexible number of trainable parameters, such that performance comparable to fine-tuned models can be achieved using a fraction of fine-tuning parameters (24, 32). Therefore, prefix-tuning can substantially reduce training time and memory cost. We implement prefix-tuning based on P-Tuning v2 (32), which applied prefix-tuning for natural language understanding tasks (e.g., classification) using RoBERTa as its pretrained backbone. For our task, we adapt P-tuning v2 by discarding its classification layer and extract QR-level embeddings by averaging the output word embeddings from its last layer. To the best of our knowledge, our work is among the first to apply parameter-efficient tuning for modeling medical text.

Built on the above modeling, for an input QR pair *s*_*n*_ of length *m*_*n*_, the prefix-enhanced QR encoder $E^{pt}$ generates a fixed-length embedding ${\text{e}}_{n}^{pt}$:


(3) $$\begin{array}{l} E^{pt}:{\mathbb{R}}^{m_{n}\times d_{model}}\to {\mathbb{R}}^{d_{model}} \end{array}$$

#### 2.3.2. General-purpose encoding via sentence transformer

Given training data, maximizing a model's learning capacity for performance and maximizing its generalizability on unseen data is a bias-variance trade-off. An alternative way to limiting the risk of overfitting a pretrained encoder on a target dataset is by using it as is, making no modification to the pretrained network. Such usage of a pretrained encoder can be thought of as a general-purpose feature extractor. We hypothesize that general-purpose embeddings can be complementary to depression-adapted embeddings.

**Figure 5:**
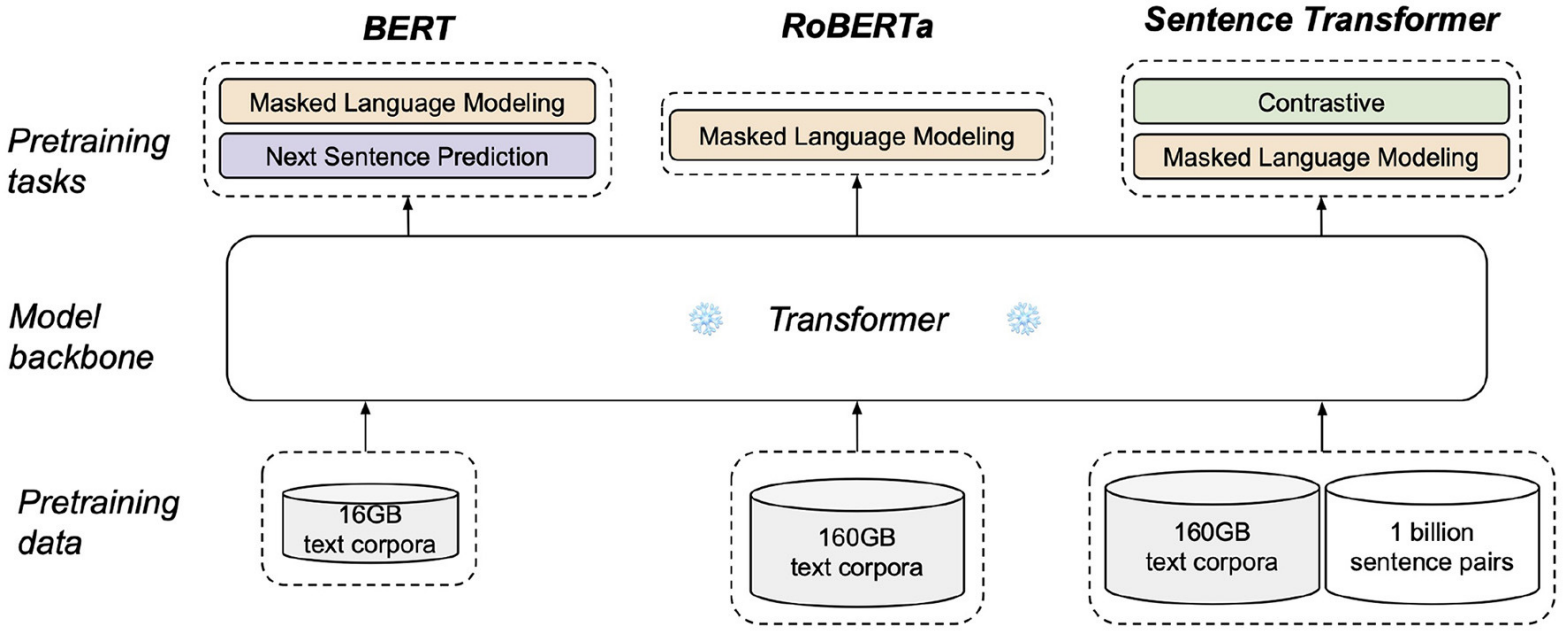
Difference in the pretraining process of BERT (*bert-base*), RoBERTa (*roberta-base*), and sentence transformer (*all-mpnet-base-v2*).

Transformer-based architecture is arguably the most popular backbone used in pretrained encoders, of which there are two factors that can affect the quality of an encoder's embedding: the pretraining data and pretraining objectives. Considering that an input QR utterance are a string of sentences, we argue that pretrained models such as BERT and RoBERTa by themselves are not the most suitable for our task as they were not pretrained for sentence-level embeddings. Although previous work have successfully applied pretrained BERT directly to encode spoken text for depression and Alzheimer's disease assessment tasks (19, 33, 34), pretrained BERT has been found to generate subpar sentence-level embeddings that are often worse than averaging non-contextualized word embeddings (35). This is likely due to BERT's pretraining objectives of masked language modeling and next sentence prediction tasks, neither of which constrained the encoder to directly optimize for generating quality sentence-level embeddings. A similar argument applies to RoBERTa, as seen in Figure 5.

Instead, we employ *all-mpnet-base-v2* from *SentenceTransformers* (ST) library (35) as a general-purpose QR encoder, $E^{st}$. ST is a framework designed to pretrain transformer-based encoders specifically for sentence-level embeddings. Specifically, *all-mpnet-base-v2* is an extension of MPNet (36) which was pretrained on the same data as RoBERTa and also architecturally faithful to BERT and RoBERTa. As illustrated in Figure 5, the ST model is additionally pretrained with a self-supervised symmetric cross entropy contrastive loss (37) on more than one billion sentence pairs. This objective constrains the encoder to produce similar embeddings for semantically similar sentences. ST models were evaluated on a number of semantic search tasks and sentence embeddings tasks. In particular, *all-mpnet-base-v2* has achieved the highest average score across 20 evaluation datasets and is thus suitable as a general-purpose encoder.

For an input QR sentence pair *s*_*n*_ of length *m*_*n*_, the general-purpose QR encoder $E^{st}$ generates a fixed-length embedding, ${\text{e}}_{n}^{st}$:


(4) $$\begin{array}{l} E^{st}:{\mathbb{R}}^{m_{n}\times d_{model}}\to {\mathbb{R}}^{d_{model}} \end{array}$$

#### 2.3.3. Fusion of depression-adapted and general-purpose embeddings

Since the two types of QR embeddings convey complementary information, we expect that making both available to the model would be beneficial. Therefore, we train our proposed model by combining **e**^*pt*^ and **e**^*st*^ at the QR level, as shown in Figure 2, denoted as dual encoder model. Three types of fusion methods are experimented: fusion by *addition, averaging*, and *concatenation*.

### 2.4. Interview-level modeling: BiLSTM with attention

The purpose of QR-sequence encoding is to capture depression-salient information over the whole interview. We treat the QR embeddings of *S* as dependent events, wherein adjacent QR pairs are likely to be related and contextually contiguous. We employ a bidirectional long-short term memory (BiLSTM) (38) layer for the sequence modeling. Although transformer can model sequences as well, it requires a significant amount of training data to be trained from scratch. Accordingly, we use BiLSTM as the annotated training data is scarce.

Specifically in our model, under the assumption that certain segments of a clinical interview are more revealing of the subject's depressive state, we endow differential attention along the sequence of QR embeddings by means of an attention mechanism. For instance, cool-down dialogues toward the end of DAIC-WOZ interviews are less likely to be relevant for depression screening than ones regarding the subject's medical history. The attention mechanism allows the model to automatically weigh and attend to the most informative QR embeddings in *E*. Formally, we assume a BiLSTM network with a single layer, with the forward and backward LSTMs computing hidden states, ${\vec{\text{h}}}_{n}$ and ${\overset{⃖}{\text{h}}}_{n}$, respectively, at every time step. Our implementation of attention is based on (39), which introduces a QR-level context vector **c** that learns to measure the relative importance of each QR pair:


(5) $$\begin{array}{ll} H_{a} & =\text{tanh}\left(W_{a}\left(H_{o}\right)\right) \end{array}$$

(6) $$w=\text{softmax}\left(H_{a}^{⊤}\cdot c\right)$$

(7) $$a=H_{o}^{⊤}\cdot w$$

where matrix *H*_*o*_ comprises the forward-backward hidden states from all time steps and is mapped to *H*_*a*_ through weight matrix *W*_*a*_. The context vector **c** is randomly initialized and learned during training. We can think of **w** as a vector of normalized attention weights, of which each weight assigns the importance of the *n*-th QR embedding. Finally, latent vector **a** is computed as a weight sum of the QR representations, effectively encoding, and summarizing the interview. This interview vector **a** is passed through a linear feed-forward layer to compute the depression severity prediction score ŷ.

### 2.5. Baseline models

To evaluate the effectiveness of our proposed model, we implement baseline models with different QR-level encoding methods for comparison. First, a prefix-only model that uses only $E^{pt}$ for QR encoding is implemented (415K tunable parameters). Given a sequence of QR pairs *S*, this model only computes depression-specific QR embeddings. Second, a ST-only model using only $E^{st}$ for QR encoding is implemented (231K tunable parameters). This model only computes general-purpose embeddings and thus might neglect linguistic semantics specific to depression-centric spoken text. Third and forth, we implement two models using a pretrained BERT and a pretrained RoBERTa, denoted as BERT-PT and RoBERTa-PT respectively, for QR encoding (231K tunable parameters). BERT is a transformer-based model that was pretrained on a large collection of English books and Wikipedia pages. RoBERTa is architecturally faithful to BERT and further improves upon BERT by modifying key training hyperparameters, substantially increasing the amount of pretraining data, and removing the next sentence prediction objective. These modifications have been empirically demonstrated to provide substantial improvements over the original BERT results on a number of language tasks. These two models serve as baselines for obtaining general-purpose QR embeddings. Lastly, four baseline models for acquiring depression-adapted QR embeddings are developed by fine-tuning pretrained BERT and pretrained RoBERTa. Given the limited training data, we restrict fine-tuning to the last transformer layer, providing models BERT-FT1 and RoBERT-FT1 (7.32M tunable parameters) and to the last two layers resulting in models BERT-FT2 and RoBERTa-FT2 (14.4M tunable parameters).

### 2.6. Data preprocessing

For replicability, below we describe the details of our data preprocessing. We first standardized the annotations throughout the transcriptions (e.g., {〈laughter〉, [laughter]} → *laughter*, {〈sigh〉} → *sigh*). Annotations irrelevant to the dialogues were removed, e.g., unique identifiers related to hardware syncing ([syncing], [sync], 〈sync〉), scrubbed dialogue due to privacy concerns (scrubbed_entry), unintelligible utterances (xxxx). Underscores in words denoting acronyms were removed (e.g., l_a → la) and all transcriptions were lowercased. Punctuation marks were kept as these could provide the models semantic information. We identified and removed interview prompts that were deemed redundant, for they were routine questions that preceded and concluded every interview (e.g., “hi i'm ellie thanks for coming in today…,” “goodbye”). After the cleaning of transcriptions, each interviewer's question and its corresponding response from a subject were concatenated as model input *s*.

### 2.7. Training strategy and implementation details

Following previous work and the original challenge's setup, we use a train-validation-test scheme on the data partitions (described in Section 2.1) to evaluate our models^2^. The development (validation) set is used to select and validate the best hyperparameters for each model, specifically by observing the loss on the development set. Performance on the development set is also used to gauge the effectiveness of each fusion method. Once the hyperparameters of a model have been determined, the model is trained five times by randomly varying the initialization seed. The mean and standard deviation of the development set performance from these five runs are reported, from which the best one is selected to be evaluated on test set.

We implemented all variants of BERT, RoBERTa, and ST using Huggingface (40) library and thus adopt the Huggingface model nomenclature. We implemented both pretrained and depression-adapted BERT and RoBERTa with *bert-base-uncased* (*L*=12, *I*_*head*_=12, 110M parameters) and *roberta-base* (*L*=12, *I*_*head*_=12, 125M parameters), respectively. P-tuning v2, which is built on top of *roberta-base*, is retrofitted from the original code^3^ to obtain sentence-level embeddings. We set the prefix length to 10 and did not find a reparameterization of the prefix vectors to be necessary as suggested by Liu et al. (32). The following hyperparameters were common to all models. Batch size was set to 2. Models were optimized through the minimization of a mean squared error loss function for the regression task of depression severity prediction. AdamW (41) optimizer was used with learning rate set to 3 × 10^−4^. Training was performed with a maximum of 200 epochs and was stopped only if the validation loss did not decrease within 20 epochs. The BiLSTM module was implemented with one layer with a hidden size of 64. All models utilized the same interview-level encoder (BiLSTM + attention) configuration which was fully tunable. During preliminary experimentation, we found that inserting a linear down-projection layer after each sentence encoding stage helped stabilize training with an additional benefit of reducing training parameters. This down-projection layer was set to 128 in size. A dropout layer with an activation probability of 0.5 was inserted before every linear layer to mitigate overfitting. All models were implemented using PyTorch (42) framework.

## 3. Results

We followed the original challenge (9) and previous work by using root mean square error (RMSE) and mean absolute error (MAE) to evaluate the regression task of depression severity prediction:


(8) $$\begin{array}{ll} RMSE & =\sqrt{\frac{1}{\left|Y\right|}\overset{\left|Y\right|}{\underset{i=1}{\sum }}{\left(y_{i}-\hat{y_{i}}\right)}^{2}} \end{array}$$

(9) $$\begin{array}{ll} MAE & =\frac{1}{\left|Y\right|}\overset{\left|Y\right|}{\underset{i=1}{\sum }}|y_{i}-\hat{y_{i}}| \end{array}$$

where |*Y*| is the number of training samples and *y*, ŷ denote the PHQ-8 ground-truth label and prediction, respectively.

### 3.1. Prediction performance of pretrained models

Results from the pretrained models are presented in Table 1 under five-run validation and test setting. The results show that the ST-only model performed better than both BERT-PT and RoBERTa-PT. We noticed that BERT-PT performed better than RoBERTa-PT on average on the development set but fell short on the test set. Compared to the two baseline pretrained models, ST-only delivered a much lower variance while achieving better results on both the development and test sets. The relatively high variance of BERT-PT and RoBERTa-PT was in part due to certain runs failing to converge within the prescribed training epoch.

**Table 1:** Development set and test set results of pretrained models (mean and standard deviation are shown for five-run validation results).

**Model**	**Development**			**Test**	
	**Loss**	**RMSE**	**MAE**	**RMSE**	**MAE**
BERT-PT	26.29 ± 9.45	5.07 ± 0.84	4.16 ± 0.75	6.10	5.01
RoBERTa-PT	29.24 ± 13.97	5.29 ± 1.26	5.29 ± 1.27	5.82	4.74
ST-only	**18.16** **±0.89**	**4.26** **±0.10**	**3.22** **±0.11**	**5.32**	**4.37**

The best score of each metric is boldfaced.

### 3.2. Prediction performance of depression-adapted models

Table 2 shows the results for the depression-adapted models. The prefix-only model performed the best on the development set with the lowest performance variance, as well as on the test set. Between the two types of proposed QR encoding methods, we highlight the performance lead of the prefix-only model over the pretrained ST-only model, albeit that the prefix-only model was not pretrained on a sentence level. This performance gain is likely due to the depression-specific knowledge gained from learning on the DAIC-WOZ dataset. Among the baseline depression-adapted models, both BERT-FT1 and RoBERTa-FT1 performed better than their two-layer fine-tuned counterparts, on both the development set and test set. Although RoBERTa-FT1 performed worse on average than BERT-FT1 on the development set, it achieved better performance on the test set.

**Table 2:** Development set and test set results of depression-adapted models (mean and standard deviation are shown for five-run validation results).

**Model**	**No. of parameters tuned**	**Development**			**Test**	
		**Loss**	**RMSE**	**MAE**	**RMSE**	**MAE**
BERT-FT2	14.4M	30.38 ± 11.79	5.43 ± 1.06	4.41 ± 0.99	6.53	5.64
BERT-FT1	7.32M	25.37 ± 5.68	4.81 ± 0.55	3.89 ± 0.37	5.72	4.76
RoBERTa-FT2	14.4M	41.97 ± 2.72	6.48 ± 0.22	5.37 ± 0.27	6.13	5.20
RoBERTa-FT1	7.32M	28.34 ± 8.74	5.28 ± 0.77	4.17 ± 0.76	5.62	4.66
Prefix-only	**415K**	**16.19** **±1.22**	**4.02** **±0.15**	**3.24** **±0.20**	**5.02**	**4.17**

The best score of each metric is boldfaced.

### 3.3. Prediction performance of dual encoder model

**Figure 6:**
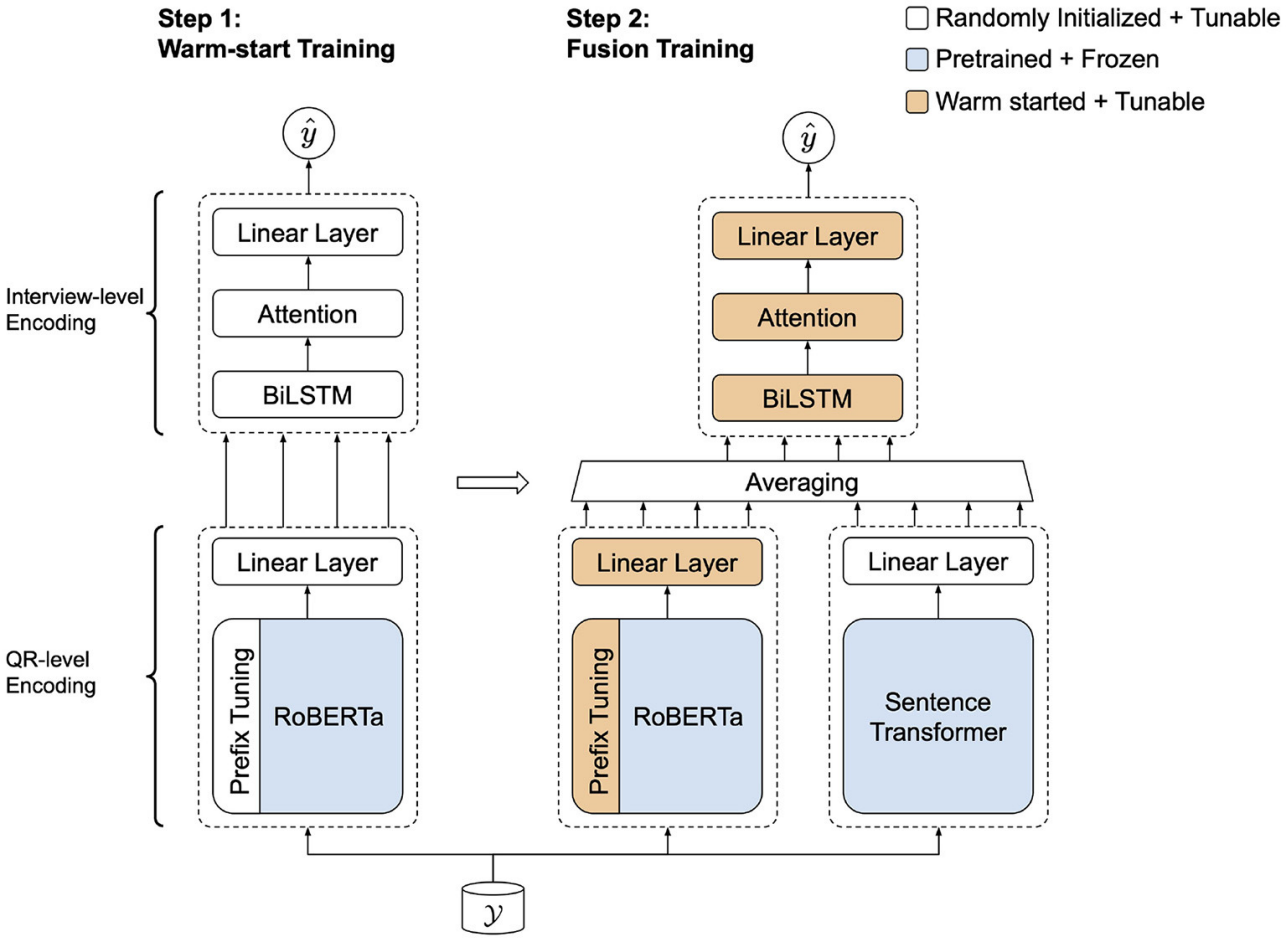
Two-step warm-start training of the proposed dual encoder.

The results of the three fusion methods are shown in Table 3. Based on the loss values, the results demonstrate that fusion by averaging performed the best among the three types of fusion methods on the development set but with a higher variance. Therefore, we used average fusion in our final implementation of the dual encoder. Motivated by the prefix-only model's slight performance gain over ST-only, we performed *warm-start* training, wherein the fusion model parameters (prefix-based encoder linear, BiLSTM, and attention layers) were initialized by copying from a previously trained model, in this case the prefix-only model. This training process can be seen in Figure 6. To verify the proposed model's performance, we performed significance testing between the warm-start dual encoder and the baseline models in Tables 1, 2. Pairwise comparisons were done using a one-way ANOVA test on the test set predictions. Both absolute errors and squared errors were tested and we found that the dual encoder was significantly better than all baseline models (*p* < 0.05).

**Table 3:** Development set results of dual encoder with three fusion methods (mean and standard deviation are shown for five-run validation results).

**Fusion Method**	**Loss**	**RMSE**	**MAE**
Concatenation	15.68 ± 0.75	3.96 ± 0.09	3.05 ± 0.18
Addition	16.12 ± 0.93	4.01 ± 0.12	3.11 ± 0.06
Average	15.46 ± 0.98	3.93 ± 0.12	3.10 ± 0.15
Average (with warm-start)	**14.02** **±1.01**	**3.74** **±0.14**	**2.96** **±0.17**

The best score of each metric is boldfaced.

Table 4 compares our models on the DAIC-WOZ dataset to the existing state-of-the-art models with test set results: the AVEC 2017 audio-and-video-based model (9), a random forest model using handcrafted text-based features (43), a speech-only attention-based model (18), and multimodal models that utilized all available audio, video, and text information (44–47). On both the development and test sets of DAIC-WOZ, all four of our text-based models outperformed the AVEC 2017 model. Using all available input modalities (audio, video, and text), the model proposed in Yang et al. (47) achieved the best results on the development set but fell short on the test set, compared to the prefix-only model and dual encoder model. On the test set, our dual encoder model achieved an improvement of 6.22% (4.67 vs. 4.98) in RMSE and 1.8% (3.80 vs. 3.87) in MAE over the best reported result (43) on the test set of DAIC-WOZ. To the best of our knowledge, our proposed model is the best performing model on this dataset, outperforming all previously reported models. While incorporating other modalities into our proposed framework is interesting, in this work our main goal is to leverage the most recent advancement in transfer learning based on large language models and parameter-efficient tuning, which are still relatively underdeveloped in other modalities due to the lack of both quality pretrained models and their corresponding parameter-efficient tuning mechanisms. However, we showed that our models have brought forward the state-of-the-art performance and we will leave the fusion of modalities as future work.

**Table 4:** Key results from current literature, experiments on baseline models, and the proposed model, in terms of RMSE and MAE scores (lower is better).

**Methods**	**Modality**	**Development**		**Test**	
		**RMSE**	**MAE**	**RMSE**	**MAE**
**Previously reported**					
AVEC2017 Baseline (9)	SV	6.62	5.52	7.05	5.66
HATN&HAE (18)	S	3.68	2.87	5.51	4.20
RF (43)	T	4.97	3.66	4.98	3.87
Topic (44)	SVT	3.54	2.77	4.99	3.96
SVM (45)	SVT	4.43	3.22	5.11	3.98
DCNN-DNN (46)	SVT	4.65	3.98	5.97	5.16
Hybrid (47)	SVT	**3.09**	**2.48**	5.40	4.36
**Proposed**					
ST-only	T	4.10	3.03	5.32	4.37
Prefix-only	T	3.76	2.92	5.02	4.17
Dual encoder	T	3.67	2.81	4.84	3.98
Dual encoder (warm-start)	T	3.56	2.79	**4.67**	**3.80**

Dual encoder achieved a new state-of-the-art performance among text-only models and multi-modal models. The best score of each metric is boldfaced (S, speech; V, video; T, text).

### 3.4. Effects of small data on depression-adapted models

To further investigate the efficiency (number of learnable parameters used) and effectiveness (prediction performance) of prefix-tuning in low-data setting, we conducted an experiment to understand the relationship between training set size and model performance. Training data was randomly sampled to produce subsets with {20, 40, 60, 80%} of the total size, separately with respect to the depressed/non-depressed groups to preserve PHQ-8 score distribution. Three models (prefix-only, BERT-FT1, and RoBERTa-FT1) were adapted for each subset, with each model trained using three random seeds. The same training subset data was applied across all models to be compared. Prefix length was a hyperparameter with possible lengths of {2, 4, 6, 8, 10} and was chosen based on a single validation run on the development set.

**Figure 7:**
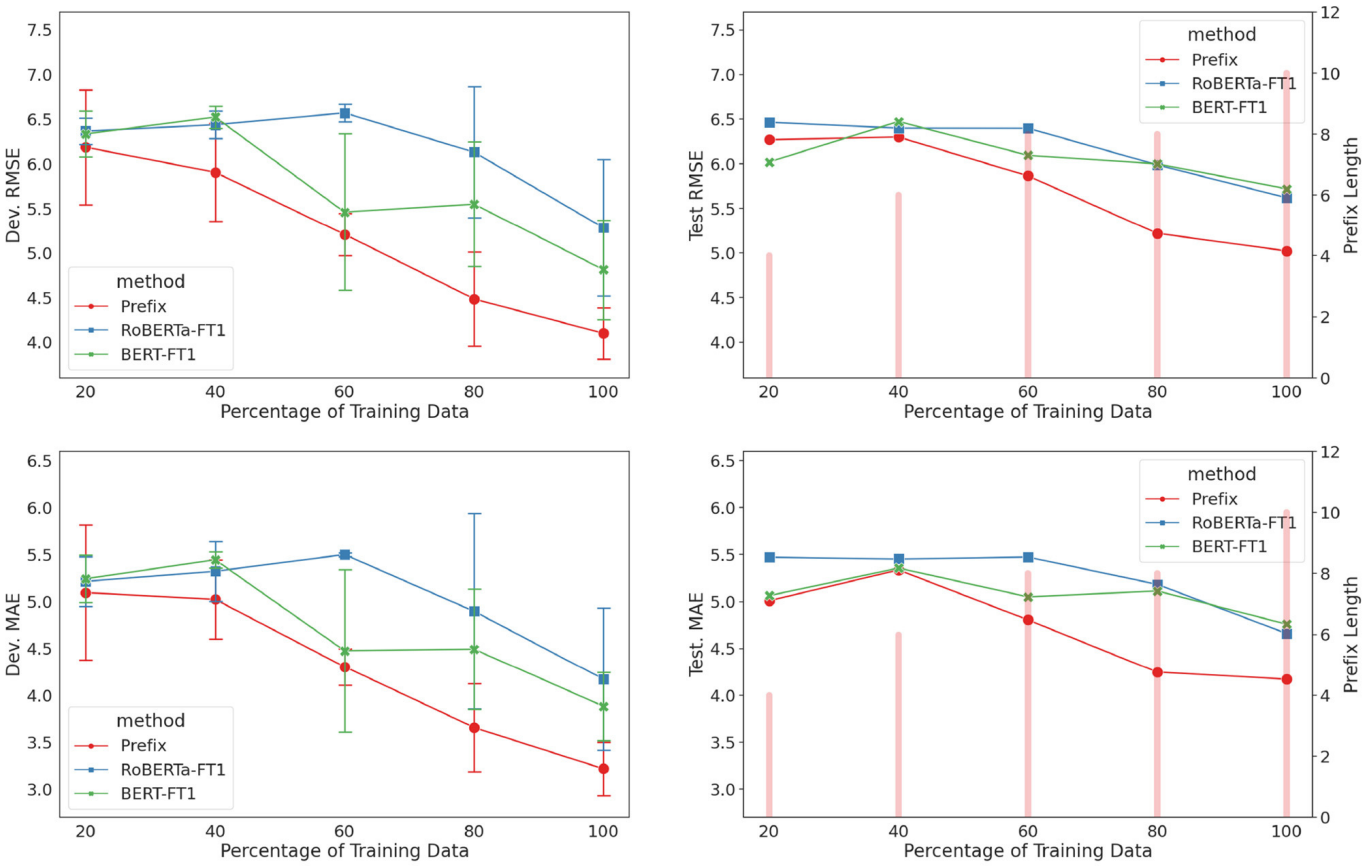
Results for low-data setting experiment (prefix-only, BERT-FT1, and RoBERTa-FT1). **(Left)** Development set results with error bars indicating standard deviation across three runs for training subset {20, 40, 60, 80%}. **(Right)** Test set results using the best model from the three runs. Prefix length for each percentage is displayed as a bar.

The results of the experiment are shown in Figure 7. All three models performed competitively at 20% with prefix-only model on average taking a slight lead on the development set. As the training set size increased, the performance gap between the prefix-only model and fine-tuning models began to widen. All three models achieved their best results when 100% of the training data was available, with the prefix-only model achieving the lowest errors on both the development and test sets.

## 4. Discussion

### 4.1. Selecting appropriate pretrained models and adaptation method

With the difference in pretraining objectives and datasets, ST-only outperformed BERT-PT and RoBERTa-PT by a significant margin (over 8% in both RMSE and MAE). BERT-PT and RoBERTa-PT were not pretrained to generate semantically meaningful embeddings for encoding sentences. On the other hand, by selecting more appropriate pretraining objectives and materials to pretrain sentence embeddings, the resultant model eased our task. Compared to the pretrained model baselines (BERT-PT and RoBERTa-PT), training of the ST-only model was more stable and converged faster. The results suggested that sentence transformer was the appropriate pretrained general purpose model for our depression severity estimation task.

While fine-tuning improves upon its pretrained counterpart (e.g., BERT-FT1 vs. BERT-PT, RoBERTa-FT1 vs. RoBERTa-PT), as an adaptation method it does not appear to be sufficient enough to surpass selecting an appropriate pretrained encoder, such as a ST-based model. Nonetheless, prefix-only outperforming ST-only suggests that selecting an appropriate adaptation method benefited depression severity prediction more than the sentence embedding pretraining.

### 4.2. Finding the right balance between pretraining and depression adaptation

We observe that pretrained models with more tuned parameters (BERT-FT2 and RoBERTa-FT2) performed worse on the development set than their less parameter-tuned counterparts (BERT-FT1 and RoBERTa-FT1). When we consider the results from both pretrained regime and fine-tuning regime, results from both the development and test sets show a trend wherein BERT-/RoBERTa-FT1 performed the best, followed by the pretrained BERT/RoBERTa, and BERT-/RoBERTa-FT2 last. On this dataset, the optimal point seems to lie between no fine-tuning and fine-tuning two layers. Results of the prefix-tuning model is fitting: using only 6% of trainable parameters compared to FT1 models (~7M parameters), the prefix-only model outperformed all other depression-adapted baselines and all pretrained-only baselines on both data partitions. This result renders evidence showing that fine-tuning on a dataset of this size would result in overparameterization, a phenomenon that has also been observed in other tasks with similar sizes of data (25). Furthermore, the performance gain of prefix-only model over ST-only model suggests that some degree of task adaptation is beneficial and reemphasizes that the method of adaptation can also be significant. This likely indicates that certain depression-specific information in the dataset can be learned by the model.

By the same token, the complementary effect observed in the dual encoder model from fusing embeddings can be seen as another approach to balancing between pretraining and task adaptation. While the RoBERTa-based prefix-tuning module was pretrained for subword-level embeddings, sentence-level embeddings from the ST encoder could be adding another layer of complementarity by providing sentence-level semantics. Furthermore, warm-start further improves model training by starting training from a better initialization point on the loss surface and converge to a better solution. Final training of the dual encoder model incorporated network parameters of the prefix-only model. This enables the ST embeddings to offer useful complementary information to ensure the resultant model to be either as good as the prefix-only model or better. Since the warm-start initialized dual encoder model has already learned some useful features from the target data, the ultimate fusion model did not have to learn them from scratch. In essence, warm-start training of the fusion model helped to reduce the amount of data and computational resources required to train a model. Thus, this training step was complementary to learning under limited training data for the fusion model. We observe that the combination of prefix-based and ST encodings improves upon the two embeddings used alone. This validates our assumption that the two types of embeddings offer complementary information to the predictive model.

While the multi-modal model from (47) achieved the best performance on the development set, their model underperformed on the test set compared to our model. The result suggests that prefix-tuning of a well pretrained model is less prone to overfitting and promotes generalizability. Interestingly, our dual encoder model which learned solely from text-based features attained the best results among all published models (including multi-modal ones). The inclusion of speech modality to complement our current model with paralinguistic information may further improve prediction performance, as evident in the referenced models in Table 4. However, as discussed above, our main goal in this work is to investigate the most recent advancement in transfer learning based on large language models and parameter-efficient tuning, and we will leave the fusion of modalities as future work.

### 4.3. Effectiveness of prefix tuning for depression adaptation

The performance curves in Figure 7 dovetail at 20% training set size. There probably exists a limit in how much information the models could learn from only so few examples (21 data points), which could not even cover the whole PHQ-8 range. As more training data points were introduced, the prefix-only model showed a greater proclivity toward lowering its prediction error, which suggests its efficiency in utilizing the new training data. On the other hand, the fine-tuning models struggled to minimize their errors. We attribute this performance gap partly to the flexibility of adjusting the prefix length, which allowed a finer adjustment to the model's learning capacity. This was evident by increase in prefix length as the amount of training data increased.

### 4.4. Limitations of automated systems

Although this work aims to address the problem of data scarcity that handicaps building ML models for healthcare, effort should still be made to expand depression datasets to increase their diversity, in order to better represent the population. Furthermore, as mentioned in Section 2.1, the PHQ-8 score range is further categorized into five severity ranges, each of which spans four points. Although our proposed dual encoder system was able to achieve a new state-of-the-art performance result on the test set of DAIC-WOZ, the performance is only just within a severity range, i.e., MAE of 3.80 comparable to a four-point range span. A difference in estimating a whole depression level could result in substantial change in treatment. There remains a sizable performance gap before achieving a clinically significant automated system. Our results demonstrate that, similar to self-assessment tools like PHQ, the ML models can only be used in support of a clinician's diagnosis by providing them an auxiliary input.

### 4.5. Text-based systems vs. speech-based systems

**Figure 8:**
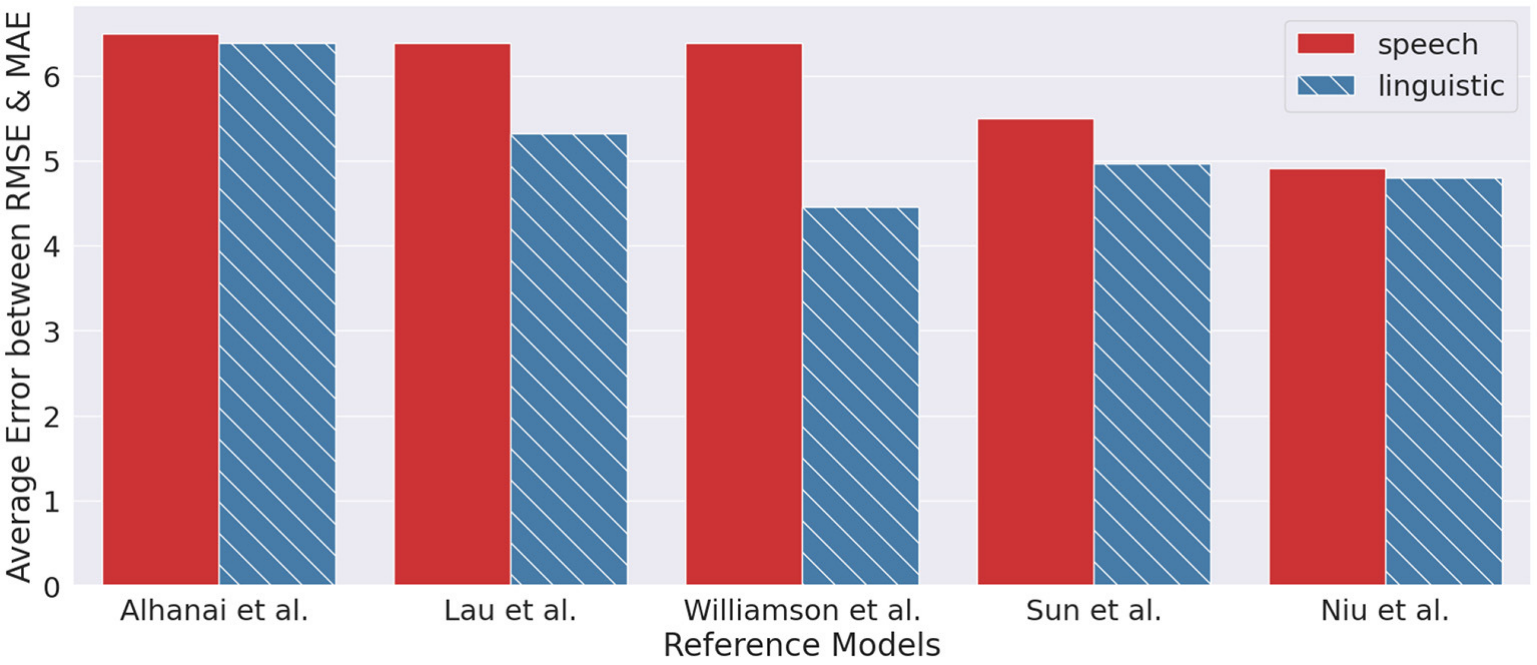
Results from reference models (20, 43, 48–50) between text-based approach and speech-based approach.

From an information theory perspective, the extraction of linguistic information from speech can be seen as information reduction, by subtracting paralinguistic information embedded in speech signals. Consequently, it is logical to presume that a model with access to the speech signal would be more predictive than a model with only the textual information. However, we observe from the current literature that the contrary is true, as seen in Figure 8, where text-based models are generally better than their speech-based counterparts. Likewise from our observation of the dual encoder model outperforming the speech-based model from (18).

We offer several reasons for why a text-only ML model may be better than a speech-only model. One reason is that text-based data is typically easier to work with than speech data. Transcribed data can be easily cleaned and preprocessed, while speech signal may be plagued with background noise, reverberation, and level differences. Another reason is that text-based models can attend to exploiting the rich context and structure of natural language; they can focus on the semantics of the spoken content by leveraging and adapting the state-of-the-art pretrained language models. Even though semantics are embedded in the speech signal, the signal carries many other types of variabilities, making it difficult for speech-only models to tease out the more task-beneficial variabilities from the irrelevant ones. Finally, speech-based models present other challenges such as reduced user privacy, where text transcript is easier to anonymize than the speech signal.

## 5. Conclusion

In this paper, we demonstrated the usefulness and effectiveness of deep pretrained language models based on a parameter efficient tuning method, prefix-tuning, for the task of depression severity prediction. Through experiments on the benchmark dataset, DAIC-WOZ, we showed that with 94% fewer training parameters, prefix-tuning outperformed models trained with fine-tuning. Furthermore, we presented a novel approach of fusing embeddings extracted from a prefix-based depression-adapted encoder and a general-purpose encoder, to enable achieving a new state-of-the-art performance in predicting the severity of depression, outperforming models that used multiple modalities. We attribute the complementarity between the two types of embeddings to their scope (depression adapted vs. general purpose) and pretraining strategies (word-level vs. sentence-level). We conducted additional experiments to understand the effectiveness of prefix-based adaptation for depression data and attributed it partly to the flexibility of optimizing the prefix length for a given amount of training data. Our research provides evidence showing that prefix-tuning is a useful and powerful approach and contributes to the development of effective tools for automatic assessment of depression severity in patients.

## Data availability statement

The original contributions presented in the study are included in the article/supplementary material, further inquiries can be directed to the corresponding author.

## Author contributions

CL contributed to the development and training of the machine learning models, to the analysis of the results, and to the writing of the manuscript. XZ and W-YC contributed to the design of the research, manuscript preparation, and supervised the findings of this work. All authors contributed to the article and approved the submitted version.

## Footnotes

^1^We ignore indices for the transformer layer and the self-attention head for brevity sake.

^2^Code of our proposed model will be made publicly available at https://github.com/clintonlau/dual-encoder-model.

^3^ https://github.com/THUDM/P-tuning-v2
